# Classical Transient Receptor Potential 1 (TRPC1): Channel or Channel Regulator?

**DOI:** 10.3390/cells3040939

**Published:** 2014-09-29

**Authors:** Alexander Dietrich, Meike Fahlbusch, Thomas Gudermann

**Affiliations:** Walther-Straub-Institute for Pharmacology and Toxicology, Member of the German Center for Lung Research (DZL), School of Medicine, LM-University of Munich, Nussbaumstr. 26, 80336 Munich, Germany; E-Mails: meike.fahlbusch@med.uni-muenchen.de (M.F.); thomas.gudermann@lrz.uni-muenchen.de (T.G.)

**Keywords:** TRPC1-deficient animal model, acinar cells, skeletal muscle, immune cells, cardiopulmonary system, kidney, platelets, tumor, bone formation, neurons

## Abstract

In contrast to other Classical Transient Receptor Potential TRPC channels the function of TRPC1 as an ion channel is a matter of debate, because it is often difficult to obtain substantial functional signals over background in response to over-expression of TRPC1 alone. Along these lines, heterologously expressed TRPC1 is poorly translocated to the plasma membrane as a homotetramer and may not function on its own physiologically, but may rather be an important linker and regulator protein in heteromeric TRPC channel tetramers. However, due to the lack of specific TRPC1 antibodies able to detect native TRPC1 channels in primary cells, identification of functional TRPC1 containing heteromeric TRPC channel complexes in the plasma membrane is still challenging. Moreover, an extended TRPC1 cDNA, which was recently discovered, may seriously question results obtained in heterologous expression systems transfected with shortened cDNA versions. Therefore, this review will focus on the current status of research on TRPC1 function obtained in primary cells and a TRPC1-deficient mouse model.

## 1. Introduction

Transient receptor potential (TRP) proteins are non-selective cation channels fulfilling diverse roles as versatile cellular sensors and effectors [1]. Originally discovered in *Drosophila*, the TRP superfamily comprises 33 channels, which are divided into seven families: TRP*C* (for *c*lassical or *c*anonical), TRP*M* (for the founding member called *m*elastatin), TRP*V* (for the first cloned *v*anilloid receptor), TRP*A* (for a member with high numbers of *a*nkyrin repeats), TRP*P* (for *p*olycystins), TRP*ML* (for mucolipins) and TRP*N* (for *N*omPC-like proteins) (see [2] for the latest nomenclature). All TRP families, except TRPN channels, are also expressed in humans and more than 20 hereditary diseases in areas as diverse as neurology, cardiology, pulmonology, nephrology, dermatology and urology are caused by mutations in 11 *Trp* genes [1]. Although TRPC1 was the first cloned mammalian TRP protein and is the founding member of the TRPC family, its molecular make-up, expression, and function as a channel in a physiological setting remains mysterious. This review will summarize an increasing set of data available for TRPC1 in different cells and organs emphasizing its importance as a prognostic and/or diagnostic markers, as well as a pharmacological target for therapeutic intervention.

## 2. Basic Features of the TRPC1 Gene and Protein

**Figure 1:**
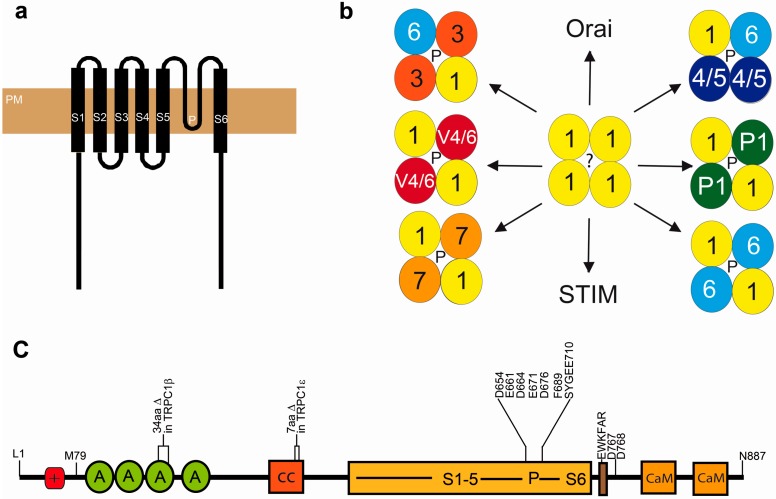
(**a**–**c**) Structural features of the extended version of TRPC1α [10]. (**a**) Topology of TRPC1 in the plasmamembrane (PM) indicating transmembane regions (S1-S6) and the predicted pore domain (P or ? for the TRPC1 homotetramer). (**b**) Heteromultimerisation potential of TRPC1. TRPC1 can interact with other members of its own family, TRPP1 and TRPV4, as well as with the proteins inducing store-operated Ca^2+^ influx: STIM and Orai. (**c**) Domain structure of TRPC1. M79 indicates the first amino acid of the short cDNA version [4,5]. +, positively charged region in the N-terminally extended protein [10]; A, ankyrin repeat; CaM, calmodulin binding site; cc, coiled coil region, D767 and 768 form the proposed STIM1 interacting site; EWKFAR, highly conserved region in the TRP box. N-terminal deletions in the functional TRPC1β [9] and TRPC1ε [10] isoforms are indicated. Essential amino acids of the pore domain (D and E) and of the selectivity filter (SYGEE) are listed. See text for more details.

After the initial discovery of transient receptor potential (*trp)* channels in the fruit fly *Drosophila melanogaster* [3], the race was on to find its first homolog in mammals. Three groups identified an expressed sequence tag (EST05093) from a human fetal brain cDNA library with high homology to the trp protein sequence [4,5,6]. The DNA and according amino acid sequence of the previously called human TRP1, a hydrophobicity plot, and the expression pattern of the protein was published in 1995 [4,5]. First functional data for TRPC1 followed in 1996 [6]. The human gene coding for TRPC1 is on chromosome 3, while the murine homologue is located on chromosome 9 [7]. The protein shows a broad but not ubiquitous expression across different cell types (reviewed in [8]). Its mRNA is expressed in five splice variants [9,10], but only three (α, β, and ε) appear to be translated and form functional proteins. While the TRPC1β isoform lacks 34 amino acids (aa) in the third ankyrin repeat, the ε isoform exposes a 7 aa deletion downstream of the first membrane spanning helix (S1, see Figure 1). The predicted sizes of α and β forms are 91 and 88 kDa, respectively. However, in native tissues, the actual size of the protein was greater than its predicted size calculated from the published amino acid sequence. This discrepancy was originally explained by its glycosylation at a predicted site between transmembrane domain 5 and the pore region [11]. Very recently however another start codon CUG was identified upstream of the published AUG resulting in an amino terminal extension of 78 amino acids [10]. This additional protein sequence harbors a cluster of positively charged amino acids [10] of still unknown function. However, it was suggested that after a conformational switch this positively charged “ball” might block the negatively charged pore domain of the channel ([12] and see Figure 1). Moreover, an additional functional splice variant TRPC1ε bearing a 34 nucleotide deletion at exon 5 was identified by the same group [10].

The putative transmembrane structure of TRPC1 can also be found in all other TRP channels cloned later and consists of intracellular N- and C-termini, six membrane-spanning helices (S1–S6), and a pore forming loop (P) with negatively charged D and E aa and a putative selectivity filter (SYGEE) between S5 and S6 (see Figure 1). Like all other TRPC proteins, TRPC1 contains an EWKFAR TRP box (EWKFAR in Figure 1c) of unknown function and a calmodulin (CaM) binding domain that partially overlaps with an IP3 receptor-binding domain [13]. Four ankyrin repeats may be important for binding of interacting proteins and the intramolecular structure of TRPC1. It was predicted that all TRP channels form tetramers like voltage gated calcium channel with a common pore in the middle (see Figure 1b). Proof of concept data were published recently for TRPV1 using electron cryomicroscopy [14].

The possible mechanosensitivity of TRPC channels, including TRPC1, is extensively discussed in the scientific community. This discussion was initiated by a report on TRPC1 in a highly mechanosensitive protein fraction from detergent-solubilized frog oocyte membranes [15]. However, mechanosensitive currents were not detectable after heterologous expression of TRPC1 or TRPC6 in African green monkey kidney (COS) or Chinese hamster ovary (CHO) cells [16]. By analysis of a TRPC1-deficient mouse model (see below) we were able to identify mechanosensitive currents in both wild-type and TRPC1-/- smooth muscle cells from cerebral arteries, which are able to sense increasing intravascular pressure to induce vasoconstriction the so called Bayliss effect [17]. Therefore, an indirect activation of TRPC channels by mechanosensitive receptors as shown for TRPC6 [18] is more likely than mechanosensitivity of the channels *per se* (summarized in [19,20]).

## 3. Molecular Make-Up and Regulation of Functional TRPC1 Channels

The molecular architecture of functional native TRPC1 channels is still a matter of debate in the scientific community. It is unclear if TRPC1 monomers alone are able to form functional channels or if the protein has only a regulatory role on channels formed by other TRP monomers. Both functions are already known for voltage gated Ca^2+^, K^+^ and Na^+^ ion channels where the channel forming units are called α-subunits while the β-subunits are only regulatory domains. However, in contrast to TRPC1, these β-subunits do not have such a high homology to the pore forming α-subunits and do not even cross the plasma membrane in complexes of voltage gated Ca^2+^ and K^+^ ion channels (reviewed in [21]). Therefore, the high similarity of TRPC1 to other TRPC channels especially in the predicted pore region would argue for a functional TRPC1 α-subunit. However, while all mammalian TRP channels form homotetramers, the translocation of TRPC1 homotetramers to the plasma membrane and homomeric TRPC1 currents in native tissues were questioned [22]. Indeed, TRPC1 coupled to fluorescent proteins was localized in the *e*ndoplasmic *r*eticulum (*ER*) [23] and intracellular vesicles [24] by confocal and *TIRF* (*T*otal *I*nternal *R*eflecting *F*luorescense) microscopy, respectively. However, it remains unclear if TRPC1 is functional in these organelles like other TRP channels (reviewed in [1]) or just on its way to the plasma membrane (see review [25]). The latter hypothesis is clearly supported by a report that was able to identify isolated TRPC1 homotetramers in the plasma membrane by atomic force microscopy (AFM) [26]. 

The properties of a native homomeric TRPC1 current is completely elusive, because even after overexpression of TRPC1 in heterologous expression systems it is difficult to obtain substantial functional signals over background recorded in untransfected cells (reviewed in [27]). After heterologous expression in HEK293 or COS cells however, the following heterotetrameric channel complexes with TRPC1 were identified in the plasma membrane: TRPC1/TRPC3 ([28,29], TRPC1/TRPC4 [22], TRPC1/TRPC5 [22,30], TRPC1/TRPC6, TRPC1/TRPC7 [29], TRPC1/TRPC4 or TRPC5/TRPC6 or TRPC7 [31,32], TRPC1/TRPC3/TRPC7 ([33] summarized in Figure 1b). It is not clear if these complexes are formed by all TRPC1 splice variants, because, recently, it was reported that the TRPC1β isoform is not able to interact with TRPC4 [34]. Most interestingly, carbachol-induced TRPC5 and TRPC4 currents changed significantly after co-expression of TRPC1 in HEK293 cells, while cells expressing TRPC1 homotetramers showed net currents which were not significantly different from carbachol-induced currents in mock transfected cells [30]. Moreover, Ca^2+^ permeabilities of HEK293 cells expressing the DAG activated homotetrameric TRPC3/6/7 channels were significantly higher than in cells co-expressing TRPC1 [29]. Transfecting cells with a TRPC1 mutant with exchanged E aa in the selectivity filter of the pore domain (see Figure 1c) results in a further decrease of Ca^2+^ permeability of TRPC1/5, as well as TRPC1/3 heterotetramers [29]. These findings clearly demonstrate that the TRPC1 pore domain is functional at least in heteromeric TRPC channel complexes. It is now highly desirable to work with the N-terminally extended TRPC1 protein [10] to test these features of TRPC1. 

TRPC1 proteins are also able to interact with proteins other than classical TRP channel monomers. Polycystin-2 (TRPP2 now named TRPP1 [2]), which is mutated in patients with polycystic kidney disease (PKD) belongs to the TRPP family of TRP protein and physically interacts with TRPC1 [35]. Two other reports confirmed the functionality of this channel complex [36] and its formation in membrane bilayers by AFM studies [37], respectively. However, the biological function of TRPC1 in the kidney remains elusive (see chapter on TRPC1 function in the kidney below). 

TRPC1 also interacts with TRPV6 belonging to the TRPV subfamily with the vanilloid receptor (TRPV1) as the founding member [38]. Ankyrin repeats of TRPC1 are essential and sufficient to suppress translocation of TRPV6 to the plasma membrane and to inhibit TRPV6 currents. Moreover, they are enough for the binding of the two proteins as revealed by *f*luorescence *r*esonance *e*nergy *t*ransfer (*FRET*) analysis of TRPC1 deletion mutants [38]. In Xenopus oocytes however, xTRPC1 did not reduce translocation of xTRPV6 to the plasma membrane but suppressed TRPV6 currents by physical interaction [39]. 

Another member of the TRPV family, TRPV4, was also reported to interact with TRPC1 [40]. The heteromeric channels were more efficiently translocated to the plasma membrane than their homomeric counterparts after emptying the internal Ca^2+^ stores [40]. Moreover, a very recent manuscript presents evidence for flow activated channels composed of TRPC1, TRPP2 and TRPV4 monomers from three different TRP families in primary cultured rat mesenteric artery endothelial cells [41].

CaM is a Ca^2+^ binding protein that not only inhibits voltage-gated Ca^2+^ channels, but also TRPC proteins. Cultured human submandibular gland (HCG) cells expressing mutant CaM unable to bind Ca^2+^ showed decreased Ca^2+^-induced channel inactivation [42]. By glutathione-S-transferase (GST) pull-down experiments two CaM binding sites were mapped to the TRPC1 protein (see Figure 1). It may be enlightening to express TRPC1 CaM deletion mutants in “knock-in” mice to evaluate the influence of CaM on TRPC1 channels in a more physiological context.

A regulation by the Homer protein is also discussed, because two binding sites in the N- and C-terminus of TRPC1 interact with Homer proteins. It was suggested that homer forms a complex with the termini resulting in inhibition of constitutive TRPC1 activity [43,44]. A similar interaction was described later for TRPV4 termini but with an involvement of a high-affinity CaM binding site [45]. 

For a summary of predicted TRPC1 interacting proteins refer to [46].

## 4. Contribution of TRPC1 to Store-Operated Ca^2+^ Entry (SOCE)

*S*tore-*o*perated Ca^2+^ *e*ntry (*SOCE*) is activated in response to the depletion of the internal Ca^2+^ stores in the ER and its functional correlate was described as a *c*alcium *r*elease *a*ctivated *c*alcium (*CRAC*) channel with unique electrophysiological properties in T-lymphocytes [47]. After cloning the TRPC1 protein and other members of the TRPC family, it was believed that TRPCs are the molecular correlate for SOCE. However, it was apparent that non-selective TRPC channel activity did not conform to the highly Ca^2+^ selective CRAC current in these cells and, for some members, namely TRPC3/6/7 *r*eceptor-*o*perated Ca^2+^ *e*ntry (*ROCE*) through activation by DAG produced by receptor stimulated phospholipases was demonstrated later (reviewed in [48]). The molecular correlate of CRAC was finally identified by cloning three four transmembrane channel proteins called Orai1-3 [49,50], which are activated by two STIM proteins (STIM1 and 2) located in the plasma membrane of the ER [51]. STIM1 and 2 contain EF-hand domains which bind Ca^2+^ in the ER lumen. Rapidly falling Ca^2+^ levels after ER depletion result in the formation of STIM aggregates called punctae, which are able to activate Orai channels after physical interaction. In several reports it was confirmed that Orai channels are pore forming unit of functional CRAC channels which are regulated by STIM proteins (reviewed in [51]). At this point, the scientific TRP community extensively discussed the possibility of a CRAC independent SOCE involving TRP and Orai channels. Indeed, some research groups reported interaction of TRPC channels, including TRPC1 with Orai and STIM proteins *in vitro* in cell lines ([52,53] reviewed in [54]), while others postulated a receptor-operated activation of TRPC channels independent of Orai and STIM [55]. However, the scientific community agrees that it is essential to dissect molecular components of non-CRAC SOCE in native cells or whole organs from appropriate gene-deficient “knock-out” mice. However, as STIM/Orai-deficient mice are embryonically lethal, a tissue and time dependent “knock-out” of these proteins is essential. 

## 5. TRPC1 Channel Function in a Physiological Setting: Lessons from a TRPC1-Deficient Mouse Model

As outlined above it is not clear, if TRPC1 monomers are pore forming α-subunits capable to assemble to functional homomeric channels or if they are able to regulate current properties of other TRPC/TRPV monomers in heterotetrameric channels as so-called β-subunits. Hopefully, the recent discovery of an extended version of TRPC1 ([10] and see above) will answer this question and will end the disputes in the scientific community raised by data using the wrong cDNA in artificial overexpression systems. Luckily, other research groups, including ours, focused on TRPC1 function in a more physiological setting in native cells, organs or even whole organisms. Along these lines, a gene deficient TRPC1-/- mouse line was established by one (AD) of us, as an attractive tool for the scientific community to test different hypothesis concerning TRPC1 function *in vivo*. Much to our surprise, most of the commercially available antibodies detected a full size band in the range of the predicted size of the TRPC1 protein from 60 to 100 kDa [56] in Western Blots of protein lysates, from both wild-type (WT) and TRPC1-/- deficient mice. Therefore, the deletion of exon 8 of the *Trpc1* gene in RNA extracted from tissues of TRPC1-/- mice were confirmed by numerous *RT* (*r*everse *t*ranscription)-PCR experiments. The construction of the mouse line and some surprising results disproving postulated TRPC1 functions in vascular smooth muscle cells were finally published 2007, seven years after its construction [17]. Although we and other research groups later confirmed the absence of TRPC1 in protein lysates from different tissues of TRPC1-/- mice with specific antibodies [57,58,59,60], we were repeatedly confronted with the argument that the protein was not deleted in the TRPC1-deficient mouse line, because some research groups used antibodies without any experiments demonstrating their specificity (see [61,62] and reply in the same issue). In the long run, however, tissues and proteins from the TRPC1-/- mouse line serve as important negative controls for the specificity of TRPC1 antibodies. An important concern using mouse lines with a constitutive deletion however, are compensatory changes in TRP and related signaling proteins. Although we compared TRPC expression in WT and TRPC1-/- mice in different tissues and found no differences except for TRPC1 [17], it will be important in the future to establish a time dependent inducible TRPC1-/- line to exclude any long term changes in the expression of related proteins. Nevertheless, we, as well as our collaborators, succeeded in identifying and publishing numerous *in vivo* functions by comparing TRPC1-deficient with wild-type (WT) mice, which are summarized in the following paragraphs of this section. 

### 5.1. TRPC1 in the Kidney

A key function of our kidneys is the disposal of metabolic end products, excess electrolytes, and water to maintain body homeostasis. To generate such a functional and efficient organ the development of the kidney proceeds through a series of different phases during embryogenesis, after birth and in adulthood. Patients with autosomal dominant PKD, however, develop cysts, which result in end stage kidney failure at 30 years of age. Mutations in three different proteins called PKD1, PKD2 and PKD3 were detected in these patients. While PKD1 is not a channel, PKD2 and PKD3 are members of the TRPP family and are now named TRPP1 and TRPP2, respectively [2]. As outlined above, TRPC1 is able to interact with TRPP1. Although the molecular mechanisms resulting in cyst formation in these patients are still elusive, a malfunction in detection of flow by the channel complex in primary cilia of kidney epithelial cells is made responsible for the disease. Most interestingly, TRPC1 is expressed in cilia [36] and was found to interact with a binding site in TRPP1, which is mutated in some PKD patients [35]. A possible physiological function of this TRP complex might be the regulation of tubular morphogenesis and maintenance of tubular structure. However, in recent reports, Ca^2+^ channels characterized in cilia from epithelial cells and mouse embryonic fibroblasts were exclusively formed by PKD1-like1 and TRPP3 (also named PKD2-like 1) channels [63] and TRPP3-/- mice showed a characteristic intestinal malrotation due to an abnormal morphogenesis [64]. Therefore, the precise function of TRPC1 in cilia of kidney cells is still unclear. 

In the proximal part of kidney nephrons, glomeruli are responsible for ultrafiltration. This process retains proteins in the blood and creates primary protein-free urine. High blood pressure, however, destroys the filtration machinery resulting in a high protein concentration in the urine called proteinuria. Glomerular mesangial cells contribute to the physiological regulation of glomerular hemodynamics by angiotensin II (Ang II)-induced contraction. TRPC1 is expressed in human and rat mesangial cells and physically interacts with TRPC4 and TRPC6 but not with TRPC3, which is also present in these cells [65]. Most interestingly, a *s*mall *i*nterfering (*si*) RNA, specific to TRPC1, was able to inhibit Ang II-induced contraction of mesangial cells *in vitro* and a TRPC1 specific antibody targeting the pore domain reduced the decline in the glomerular filtration rate (GFR) induced by Ang II [66]. If TRPC1, alone or in a heteromeric channel complex with TRPC6 and/or TRPC4, was responsible for this effect, is still unknown. Glycation of glomerular proteins by complex molecular mechanisms [67] due to high glucose levels in untreated diabetic patients is also a reason for a defective filtration process. Therefore, proteinuria is a characteristic symptom of diabetic nephropathy (DN), which results in endstage renal disease [67]. The *Trpc1* gene maps to a chromosomal region considered to be a hotspot for DN and genetic polymorphisms in the gene have been linked to DN in the Han Chinese population [68]. Moreover, a reduced TRPC1 mRNA expression was identified in diabetic rat models [69] and the transcription factor hepatocyte nuclear factor 4α, of which dysfunction is associated with diabetes, was identified as a target of the gene [69]. Although no association of TRPC1 polymorphisms with DN was found in the US population, TRPC1 mRNA levels were significantly reduced in 26-week-old db/db mice [70], which express mutant leptin receptors and are used as a mouse model for diabetes type 2.

### 5.2. TRPC1 in Acinar Cells of the Salivary Glands and the Pancreas

Regulation of fluid and protein secretion in acinar cells of the salivary glands and the pancreas are essential for their function. Increases in the intracellular Ca^2+^ concentration ([Ca^2+^]_i_) induce secretion by activating Ca^2+^ activated K^+^ channels or Cl^−^ channels in cells of the salivary glands or the pancreas (summarized in [54]). Importantly, TRPC1-/- mice showed a substantial decrease in fluid secretion from salivary glands [57]. Increases in [Ca^2+^]_i_ were significantly reduced in acinar cells of the glands from TRPC1-deficient mice compared to cells from WT mice, although Orai channels are also expressed. Similarly, acinar cells from TRPC1-/- pancreas show reduced [Ca^2+^]_i_ levels after stimulation. A possible explanation for these findings is the specific localization of the channels in these cells. While Orai localization is restricted to the apical pole of the lateral membrane, TRPC1 is expressed in apical and basolateral regions [71]. STIM1 proteins relocate to regions of the cells containing both channels and may also activate TRPC1 in acinar cells of the pancreas [54,71]. 

### 5.3. TRPC1 in Skeletal Muscle Function and Development

In skeletal muscle ryanodine receptors and voltage-gated L-type Ca^2+^ channels are responsible for its key function: excitation contraction coupling. TRPCs as non-voltage gated Ca^2+^ channels however, play an important role in skeletal muscle differentiation and in patients with an inherited muscle dysfunction called *D*uchenne *m*uscular *d*ystrophy (*DMD*). TRPC1 is expressed in skeletal muscle [72], myoblasts and myotubes [73,74]. It was demonstrated that myoblasts expressing TRPC1 specific *s*mall *h*eterogenous (*sh*) RNAs migrate and fuse into myotubes with reduced speed in comparison to cells expressing the control shRNA [75]. The induced Ca^2+^ influx through TRPC1 homo- or heterotetrameric channels induces a transient activation of the Ca^2+^ dependent calpain proteases, which proteolyze *m*yristoylated *a*lanine *r*ich *C*-*k*inase *s*ubstrate (*MARCKS*), allowing myoblast migration and fusion [75]. By employing the TRPC1-deficient mouse model the authors were also able to identify less myofibrillar protein, smaller fiber-cross sectional areas and increased muscle fatigue in TRPC1-/- compared to WT skeletal muscles [76]. These results were confirmed by an additional study, which also reported a reduced resting stiffness after eccentric contractions in WT mice, which was lost in TRPC1-/- mice [77]. Moreover, TRPC1-/- muscles regenerated much slower after cardiotoxin-induced muscle injury, expressed less myogenic transcription factors (e.g., myoD, Myf5 and myogenin) and showed less Akt, as well as p70S6K phosphorylation [78]. Therefore, the PI3K/Akt/mTor/p70S6K which is important for muscle regeneration is down-regulated in TRPC1-/- muscles. 

Mice expressing a defective dystrophin (mdx mice) are used as an animal model for DMD although many symptoms are less severe than in human patients. Mdx mice showed increased TRPC1 [79] or TRPC1/4 [74] activity, which results in higher proteolysis and muscle necrosis (summarized in [73]. Similar results were obtained in Homer 1-deficient mice which displayed constitutively active TRPC1 channels in their myotubes and are suffering from a myopathy [44]. Although the exact localization of TRPC1 in skeletal muscle is still a matter of debate because of controversial results with antibodies of unknown specificity ([61,62] and see above) it is believed that the channel forms a so-called “costameric” macromolecular complex in the sarcolemma containing Homer 1, dystrophin, dystroglycans and many other proteins (summarized in [73]).

### 5.4. TRPC1 in Development and Bone Formation

**Figure 2:**
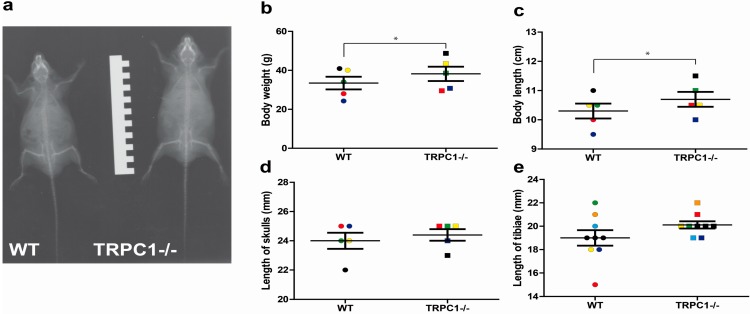
(**a**–**e)** Morphological analysis of TRPC1-/- mice in comparison to WT mice. (**a**) X-ray of TRPC1-/- and WT male littermates 10 months old (ruler marks: 0.5 cm). (**b**,**c**) Analysis of body weights (**b**) and body lengths (**c**) of 2–10 months old male littermates. Colors label littermates (*, *p* = 0.0135 and *p* = 0.0161 in a paired Student’s t-test, respectively). (**d**,**e**) Analysis of skull lengths (**d**) and tibiae lengths (**e**) in 2–10 months old male and female littermates. Colors label littermates (data are not significant different in a paired Student’s *t*-test). Lines indicate mean and standard error of the mean (SEM). Data are from the Diploma thesis of Meike Fahlbusch (Philipps-University Marburg 2008) [80].

Our initial analysis revealed heavier TRPC1-/- mice with an increased body length as compared to their WT littermates (see Figure 2) at different stages of development (two to 10 months after birth) and our collaborators identified an increased body weight in 12 to 16 weeks old mice [57]. While organ weights (heart, liver, kidney, lungs) were not significant different, three out of five skulls and seven out of nine tibiae were larger in TRPC1-/- as compared to WT mice (Figure 2). 

Bones are dynamic tissues that are constantly rebuilt by osteoblasts producing matrix and minerals for bone formation, while osteoclasts induce bone resorption. In a recent report a 14% increase in bone mass and a 27%–28% decrease in osteoclast numbers were identified in the TRPC1-/- mouse model compared to WT mice. Both values, however, were not significantly different [10]. Most interestingly, mice deficient for the *i*nhibitor of *M*yoD *f*amily *a* (*I-mfa*) had increased osteoclast numbers resulting in an osteopenic phenotype which could be rescued by mating these mice with TRPC1-/- mice. Along these lines, I-mfa deletion induced a higher Ca^2+^ influx after store depletion in myeloid precursor cells, which differentiate to osteoclasts [10]. Therefore, I-mfa may inhibit TRPC1 function and its absence may induce osteoclastogensis in I-mfa-/- mice. Moreover, mice [81] and osteoclasts [82,83] lacking the Ca^2+^ selective Orai channels showed a significantly reduced osteoclastogenesis similar but much more severe than that identified in the TRPC1-/- mouse model. These data may be the first proof for a formation of a store-operated channel with Orai as the core component and TRPC1 as a regulatory β-subunit in a physiological setting. The authors also describe an extended version of TRPC1 (see above and Figure 1) with higher Ca^2+^ selectivity than the truncated version, but only if STIM1 and Orai were co-expressed in HEK293 cells [10]. Moreover, they report that an additional TRPC1 splicing variant (TRPC1ε, see above and Figure 1) expressed in high levels in myeloid precursors and pre-osteoclasts was able to increase a highly selective CRAC current formed by Stim1 and Orai1 [10]. Although the authors were not able to provide evidence for all details, they present an interesting hypothesis for the roles of TRPC1 isoforms in concert with Orai and STIM during osteoclast differentiation. In hematopoetic cells, the extended version of TRPC1α may induce a non-CRAC SOC in a complex with Orai and next to a STIM/Orai CRAC current, while differentiation by *m*acrophage *c*olony *s*timulating *f*actor (*M-CSF*) into myeloid precursor cells results in I-mfa and TRPC1ε expression inducing a low CRAC and an I-mfa inhibited SOCE. After *R*eceptor *A*ctivator of *N*F*κ*B-*L*igand (*RANKL*) and M-CSF induced differentiation to early pre-osteoclasts I-mfa down-regulation initiates SOC and CRAC currents, which supply the cell with enough Ca^2+^ for the activation of *n*uclear *f*actor for *a*ctivated *T*-cells (*NFAT*) and other regulators of osteoclastogenesis [10]. In summary, this report presents several lines of evidence for an involvement of TRPC1 in SOCE in osteoclasts but does not answer the question if TRPC1 is also able to form heteromeric complexes with other TRP channels in these cells to induce a ROCE, activated by the described ligands essential for osteoclast differentiation.

TRPC1 expression was also described in osteoblasts, and knock-down of TRPC1 by specific siRNAs resulted in a reduction of *p*latelet-*d*erived *g*rowth *f*actor *B* (*PDGFB*)-induced proliferation [84]. In TRPC1-/- mice, a reduced number of osteoblasts were detected, which must have increased function because mineral apposition and bone formation rates were not changed as compared to WT mice [10]. The exact function of TRPC1 for osteoblast function and differentiation, however, was not yet analyzed. In summary, TRPC1-/- mice develop an increased bone mass, which is however not significant different to WT mice. Therefore, an increased body length and weight in TRPC1-/- mice in comparison to their WT littermates (Figure 2) point to an additional function of TRPC1 in growth and development which was not studied yet. 

### 5.5. TRPC1 in Platelets and Immune Cells

Thrombosis is important after injury to inhibit excess bleeding, but can also be life-threatening if a blood clot inhibits blood flow. Therefore, a tight control of platelet function during initiation and progression of thrombosis is essential for the human body. Although TRPC1 is highly expressed in platelets [85], TRPC1-/- mice display normal platelet aggregation and thrombus formation *in vivo* and *in vitro*. Moreover, no differences in Ca^2+^ entry after depletion of internal Ca^2+^ stores was detected in platelets of these mice compared to WT mice [60]. Mice expressing a constitutively active STIM1 protein (*Stim1^Sax/+^*) resulting in an elevated SOCE with macrothrombocytopenia and bleeding [86] were not rescued by mating them with TRPC1-/- mice [60]. These data exclude exclusive functional interactions of TRPC1 with STIM1 and are in clear contrast to results obtained in platelets using a TRPC1 antibody [87] of which specificity was questioned [56]. In additional experiments, different lots of this antibody failed to detect heterologously expressed TRPC1 and TRPC1-STIM1 interaction in immune-precipitation experiments in platelets [60]. However, irrespective of the proven un-specificity of this antiserum, some research groups continue to publish data confirming a TRPC1-STIM1-Orai1 interaction using this tool (e.g., [88]). STIM1-/- [89] and Orai1-/- mice [90] hardly survive the first months after birth, but platelets can be isolated and analyzed. They show severe defects in aggregation and thrombus formation [89,90], clearly confirming results of a TRPC1 independent platelet regulation.

While TRPC1 seems to be replaceable in platelets and its function in these cells remain elusive, results in an allergic mouse model indicate important functions of TRPC1 in the immune system. TRPC1-deletion resulted in a reduced production of T helper type 2 (Th2) cytokines and chemokines in the lungs of ovalbumin-sensitized mice [91]. Most interestingly, a similar phenotype was observed in TRPC6-/- mice [92], pointing to the importance of both channels for immune-regulation probably in a heteromeric TRPC1/6 channel complex. TRPC1-deficient spleens were significantly larger with a higher number of germinal centers [91]. Moreover, elevated levels of immunoglobulins in the serum of TRPC1-/- mice, in comparison to WT mice, point to abnormal B cell development and function [91], which is worth further analysis on a cellular and molecular level. Molecular insights into the regulation of cellular TRPC1 activity were recently presented. Caspase 11 was able to interact with N- and C-termini of TRPC1 and decreased TRPC1 levels after co-expression in HEK293 cells [93]. Consistent with the notion that *l*ipo*p*oly*s*accharide (*LPS*)-induced caspase 11 activates *i*nter*l*eukin *1β* (*IL1β*) secretion, a TRPC1-/- sepsis model showed increased secretion of IL1β after LPS challenge [93]. The proteolysis of the inhibitory TRPC1 activity by caspase 11 for the induction of IL1β production was very specific, because caspase 1 had no effect on TRPC1 [93]. Unfortunately, the specificity of the TRPC1 antibody used in native and transfected cells in this report [93] was also not documented. In summary, there is evidence for both immune-activating and immune-suppressive functions of TRPC1. Further studies will be necessary to evaluate the importance of TRPC1 as a drug target for immune-modulatory intervention.

### 5.6. TRPC1 and Neuronal Function

TRPC1 is expressed early in development and several reports present evidence for an involvement of its *Xenopus* homologue (XTRPC1) in axon guidance. In developing growth cones XTRPC1 was made responsible for mediating chemotropic turning in netrin and myelin-associated glycoprotein (MAG) gradients [94,95]. TRPC1-/- mice, however, do not show gross abnormalities in neuronal development (Dietrich, Fahlbusch, and Gudermann unpublished data [96]) favoring again a regulatory role for TRPC1 in heteromeric complexes with other TRPC channels. Along these lines, TRPC1 reduces migration and motility [29] in immortalized GnRH neurons [97], most probably by inhibiting Ca^2+^ permeability of heteromeric TRPC channel complexes ([29] and see above). In a more physiological setting, TRPC1-/- and TRPC4-/- mice, like WT mice, induce normal neuronal sprouting in bladders injected with cyclophosphamide, while TRPC1/4 double deficient mice exhibit no bladder innervations [98]. Cystitis with bladder over-activity and pain are long known side effects of the cytostatic drug cyclophosphamide. Normal bladder function and cystitis-induced pain however, was not altered in TRPC1/4-/- mice [98] indicating that other proteins are responsible for these functions.

TRPC1 was detected in perisynaptic regions of the cerebellar parallel fiber-Purkinje cell synapse and physically and functionally coupled with the *m*etabotropic *glu*tamate *r*eceptor *1* (*mGluR1*) to induce a slow *e*xcitatory *p*ost*s*ynaptic *c*onductance (*EPSC*) [99]. However, detection and blocking experiments in native and cultured cells were done with a TRPC1 antibody of questionable quality [56,60]. The authors reconstituted the 3,5-dihydroxyphenylglycine (DHPG)-induced currents in a heterologous expression system using CHO cells, and identified reduced currents in cells expressing a dominant negative TRPC1 channel [99], but the physiological relevance remained elusive. Therefore, our collaborators analyzed EPSC in cerebellar slices from different TRPC-/- mouse lines and found that in TRPC3-/- Purkinje cells EPSC and mGluR-mediated inward currents were completely absent, while they were unaffected in TRPC1-/- cells [100]. As an expected result of this cerebellar defect, TRPC3-/- mice exhibited an impaired walking behavior, which was not detected in TRPC1-/- mice [100].

In *d*orsal *r*oot *g*anglions (*DRG*) TRPC1 α and β isoforms are expressed and down-regulation resulted in reduced mechanosensitivity of these neurons [101]. Another report also identified TRPC6 and TRPV4 channels in DRG and decreased expression of all three channels by specific antisense oligodeoxynucleotides reduced hyperalgesia to mechanical and hypotonic stimuli [102]. Thermal injury however, was only inhibited by reduced TRPC6 and TRPV4 levels [102]. These data prompted our collaborators to study mechanical responses in sensory neurons in the TRPC1-deficient mouse line. Most interestingly, mechanically evoked action potentials were decreased in slowly adapting Aβ- and in rapidly acting Aδ-fibers in TRPC1-/- mice in comparison to WT mice [103]. In summary, TRPC1 together with other channels seems to be involved in the detection of innocuous mechanical forces, because behavioral responses to these stimuli were reduced by ~50% in these mice [103]. As outlined above, TRPC1 is most likely not mechanosensitive *per se*, but is involved in the transmission of the action potentials in the specified fibers and their processing in the DRG neurons. 

The lateral septum of the brain is highly vulnerable to seizure-induced cell death. Interestingly, the large depolarizing plateau potential which is essential for the epileptiform burst firing by metabotropic glutamate receptors was abolished in septal neurons of TRPC1/4 double deficient mice and reduced by 74% in TRPC1-/- mice compared to WT mice [104]. Therefore, TRPC1/4-/- mice did not die after pilocarpine-induced seizures, while WT mice had high mortality rates of ~70% after injection of the highest dose (280 mg pilocarpine/kg body weight), although average seizures scores were not different in both mice line [104]. Both seizure size and neuronal cell death, however, were reduced in TRPC5-/- mice [105]. These data would favor a heteromeric TRPC1/4 channel and a homomeric TRPC5 channel with distinct functions in neurons of the lateral septum.

Most interestingly, patients with Parkinson disease (PD), characterized by a loss of dopaminergic neurons in the substantia nigra, show decreased TRPC1 expression levels and higher levels of the unfolded protein in brain lysates [106]. Thus, a neurotoxin-induced mouse model for PD over-expressing TRPC1 was protected from the disease [106]. Moreover, Ca^2+^ entry through TRPC1 as a homomeric or heteromeric channel complex activates the Akt/mTOR pathway in a similar way as in skeletal muscle (see above), while loss of TRPC1 in TRPC1-/- mice or high levels of unfolded TRPC1 inhibits the neuro protective pathway [106]. Therefore, to-be-identified TRPC1-activating drugs may be helpful in slowing down PD progression.

### 5.7. TRPC1 in the Cardiopulmonary System

Cardiovascular disease is the leading course of death in industrialized countries and there are numerous sometimes-contradictory reports of an involvement of TRPC proteins in rat and mouse models simulating these pathophysiological processes (reviewed in [107,108]). 

Cardiac hypertrophy induced by pressure overload, such as chronic hypertension and aortic stenosis, are responsible for slowly progressive remodeling processes which result in a life-threatening reduction of cardiac output. TRPC1 down-regulation by specific siRNAs attenuated cardiac hypertrophy [109] and the *Trpc1* gene has conserved NFAT consensus sites in its promoter region [109]. The Ca^2+^/calmodulin-dependent serine/threonine phosphatase calcineurin dephosphorylates NFAT which is then able to translocate to the nucleus resulting in activation of hypertrophic response genes [110] (e.g., *Trpc1*). To test this hypothesis, TRPC1-/- mice were exposed to *t*horacic *a*ortic *c*onstriction (*TAC*) and neurohumoral excess by application of Ang II [58]. In contrast to WT mice, TRPC1-deficient mice fail to develop maladaptive cardiac hypertrophy. The authors propose a mechanosensitive signaling through calcineurin/NFAT and identified an altered mTOR/Akt signalling in TRPC1-/- cardiac lysates compared to WT lysates [58]. Although they identified an increased leak-like current in WT cardiomyocytes after application of TAC which is missing in TRPC1-deficient cells [58], it is rather unlikely that TRPC1 alone is responsible for cardiac hypertrophy. Other researchers also convincingly demonstrated an involvement of TRPC3 and TRPC6 (reviewed in [108]) and transgenic mice expressing dominant-negative forms of TRPC3, TRPC6 and TRPC4, which will also inhibit TRPC1 heteromeric, but not homomeric, channels are almost completely protected from cardiac hypertrophy [111]. Therefore, the unique part of each TRPC channel for the development of a maladaptive hypertrophic heart remains elusive.

Endothelial cells are specialized epithelial cells which reduce blood flow turbulence and strictly regulate the transport of liquids across the semi-permeable vascular endothelial barrier. Vascular inflammation however, induces changes in endothelial cell shape and consequently increases endothelial permeability. This mechanism is helpful for invading immune cells, but can also induce life-threatening edema formation in several tissues including the lung. Again several TRPC channels namely TRPC1, TRPC4 and TRPC6 were made responsible for the disruption of barrier function in pulmonary arteries yet by one research group ([112,113,114,115,116] reviewed in [108]). Because deletion of TRPC4 in TRPC4-/- mice inhibits increases in thrombin-induced lung vascular permeability only to about 50% [115], the involvement of more than one TRPC channel is obvious. Therefore, edema formation after ischemia-reperfusion in TRPC1-, TRPC4- and TRPC6-deficient lungs was quantified [117]. Much to our surprise, only TRPC6-/- lungs were completely protected from edema formation [117], while TRPC1- and TRPC4-deficient lungs developed edemas indistinguishable from WT lungs. In additional experiments, we were able to demonstrate that TRPC6 function in endothelial cells but not in immune cells were responsible for this pathophysiological process [117], which represents a serious challenge in lung transplantation. Therefore, at least lung edema formation by ischemia is independent of TRPC1.

Smooth muscle cells do not only provide structural integrity for the vessel but also precise regulation of vascular tone and blood pressure. It was proposed that TRPC1 plays an important role in vascular and pulmonary vasoconstriction and smooth muscle cell proliferation (reviewed in [108]). Although overexpression of TRPC1 in rat pulmonary arteries resulted in enhanced SOCE-induced vasoconstriction [118] we did not detect differences in Ca^2+^ influx after store depletion in vascular smooth muscle cells isolated from TRPC1-/- mice, but were able to demonstrate that SOCE was exclusively dependent on STIM1 expression [17]. The same was true for a proposed role of TRPC1 in neointimal hyperplasia [119], because TRPC1-deficient mice had no reduced levels as compared to WT mice [108]. In contrast to the systemic vasculature, the pulmonary circulation responds to hypoxia by constricting pulmonary arteries and diverting blood flow to the well ventilated areas of the lung to ensure maximal oxygenation of the venous blood. Hypoxic pulmonary vasoconstriction (HPV) includes two phases, an acute vasoconstrictor response occurring within seconds to minutes as well as a sustained one developing over several hours and inducing a process of vascular remodeling after several weeks. Most interestingly, the acute HPV was completely missing in TRPC6-/- mice [120] while vascular remodeling during the chronic phase was at least in part dependent on TRPC1 expression [59]. To understand TRPC1 function on a cellular basis, *p*ulmonary *a*rterial *s*mooth *m*uscle *c*ells (*PASMC*) were isolated from the small precapillary pulmonary arteries, which are responsible for tonus regulation. TRPC1 expression was up-regulated in PASMC and proliferation of TRPC1-/- PASMC was reduced after chronic hypoxia in comparison to WT cells [59]. Our data were reproduced independently by another research group, which demonstrated reduced vascular remodeling after chronic hypoxia in TRPC1-/- mice, but, in contrast to us, also in TRPC6-deficient mice [121]. Pulmonary vascular tone was reduced in TRPC1-/- and TRPC6-/- large pulmonary arteries [121] which are however not responsible for the regulation of the pulmonary blood pressure (see above). The contradictory results in TRPC6-/- mice maybe due to differences in the mouse strains though we fully agree that not TRPC1 alone but in a heteromeric complex with other TRPC channels is responsible for chronic hypoxic vascular remodeling. The search is on in our laboratory to find the molecular components of this channel complex as important pharmacological targets for therapeutic intervention in patients with chronic pulmonary hypertension.

### 5.8. TRPC1 as a Tumor Marker

There are also a few reports describing TRPC1 expression in tumors and tumor cells raising the possibility that this protein may serve as a diagnostic marker. In human breast ductal adenocarcinoma TRPC1 over-expression together with TRPM7 and TRPM8 correlated with proliferative parameters, but only TRPV6 expression correlated with their metastatic potential [122]. In a human, glioma cell line TRPC1 was responsible for regulating EGF-induced chemotaxis [123]. Moreover, Ca^2+^ entry via TRPC1 regulates activation of voltage gated Cl^−^ channel 3 (ClC-3), which is also important for cell migration [124]. However, it remains elusive if TRPC1 is essential in metastasizing brain tumors *in vivo*. 

## 6. Conclusions 

Although it is still not clear if TRPC1 proteins work as a channel of its own in homomeric complexes or, rather, as regulatory β-subunits in heteromeric complexes, the mild phenotype of a TRPC1-/- mouse model favors the latter hypothesis. Despite to its broad expression TRPC1-/- mice are healthy and have a normal life span. In some organs however, double deficient mice display significant phenotypes (e.g., TRPC1/4-/- mice in bladder innervation [98] and epilepsy-induced neurodegeneration [104]). Experiments in a heterologous expression system indicate that TRPC1 participates in the formation of a channel pore and decreases Ca^2+^ selectivity of heteromeric TRPC channels [29] or TRPV6 specific currents in a complex with TRPV6 monomers [38,39]. This inhibitory action of TRPC1 was confirmed in a TRPC1-/- sepsis model, which secretes more IL1β [93], and may be responsible for the increased length and weights of the TRPC1-/- mice in comparison to their littermates (see above). However, all other phenotypes listed above favor a loss-of-function phenotype of heteromeric channels in TRPC1-/- mice. Recently, the situation became even more complicated when an extended form of the TRPC1 cDNA was identified [10], which may represent the real native protein much better than the truncated form used in numerous overexpression studies. Hopefully *in vitro* studies with the full-length cDNA will shed new light on TRPC1 function, because the multiple phenotypes of a TRPC1 deletion in mice point to important functions *in vivo*.

## List of Abbreviations

Aa: amino acids
Ang II: angiotensin II
AFM: atomic force microscopy
[Ca^2+^]_i_: intracellular Ca^2+^ concentration
CaM: calmodulin
CHO: Chinese hamster ovary cells
ClC-3: voltage gated Cl^−^ channel 3
COS: African green monkey kidney cells
DMD: Duchenne muscular dystrophy
DN: diabetic nephropathy
DRG: dorsal root ganglions
EPSC: excitatory postsynaptic conductance
ER: endoplasmic reticulum
EST: expressed sequence tag
GFR: glomerular filtration rate
GST: glutathione-S-transferase
HEK: human embryonic kidney cells
HCG: cultured human submandibular glands
HPV: hypoxic pulmonary vasoconstriction
IL1β: interleukin 1β
I-mfa: inhibitor of MyoD family a
LPS: lipopolysaccharide
MARCKS: myristoylated alanine rich C-kinase substrate
M-CSF: macrophage colony stimulating factor
mGluR1: metabotropic glutamate receptor 1
NFAT: nuclear factor of activated T-Cells
PASMC: precapillary pulmonary arterial smooth muscle cells
PD: Parkinson’s disease
PDGFB: platelet-derived growth factor B
PKD: polycystic kidney disease
Sh-RNA: small hairpin RNA
Si-RNA: small interfering RNA
RANKL: Receptor Activator of NFκB-Ligand
ROCE: receptor-operated Ca^2+^ entry
SOCE: store-operated Ca^2+^ entry
TAC: thoracic aortic constriction
STIM: stromal interaction molecule
TIRF: total internal reflecting fluorescence
TRPC1: Classical Transient Receptor Potential 1
WT: wild-type
XTRPC1: *Xenopus* homologue of TRPC1
